# Definition of Internal Mammary Node Target Volume Based on the Position of the Internal Mammary Sentinel Lymph Nodes Presented on SPECT/CT Fusion Images

**DOI:** 10.3389/fonc.2019.01553

**Published:** 2020-01-31

**Authors:** Xue Wang, Wei Wang, Jian-Bin Li, Zong-Wei Huo, Min Xu, Peng-Fei Qiu, Ying-Jie Zhang, Feng-Xiang Li, Jin-Zhi Wang

**Affiliations:** ^1^School of Medicine and Life Sciences, University of Jinan-Shandong Academy of Medical Sciences, Jinan, China; ^2^Department of Radiation Oncology, Shandong Cancer Hospital and Institute, Shandong First Medical University and Shandong Academy of Medical Sciences, Jinan, China; ^3^Department of Nuclear Medicine, Shandong Cancer Hospital and Institute, Shandong First Medical University and Shandong Academy of Medical Sciences, Jinan, China; ^4^Department of Breast Surgery, Shandong Cancer Hospital and Institute, Shandong First Medical University and Shandong Academy of Medical Sciences, Jinan, China

**Keywords:** breast cancer, internal mammary sentinel lymph node, SPECT/CT fusion image, internal mammary lymph node irradiation, clinical target volume definition

## Abstract

**Purpose:** Mapping the distribution of internal mammary sentinel lymph nodes (IM-SLNs) presented on single photon emission computed tomography in conjunction with computed tomography (SPECT/CT) images to explore the value of IM-SLN to guide tailored clinical target volume (CTV) delineation of postoperative prophylactic IMNI.

**Materials and methods:** Ninety-seven patients who underwent preoperative lymphoscintigraphy by SPECT/CT and had imaging of IM-SLN were selected in this study. The imaging IM-SLNs on SPECT/CT of eligible patients were projected onto corresponding anatomical positions of a representative axial CT image. The IMN CTVs were delineated on the representative axial CT images according to the Radiation Therapy Oncology Group (RTOG) and Danish Breast Cancer Cooperative Group (DBCG) guideline, and defined as CTV_RTOG_ and CTV_DBCG_. The location of the IM-SLNs was compared with the RTOG and DBCG guidelines of IMN target volume delineations, respectively. The intercostal space distribution of IM-SLNs was recorded. The distances from the CTV_RTOG_ and CTV_DBCG_ to the IM-SLNs were measured, respectively.

**Results:** The total number of imaging IM-SLNs was 136. IM-SLNs were mostly found in the first intercostal space (40.4%), with 30.2, 24.3, 4.4, and 0.7% of IM-SLNs in the second, third, fourth, and fifth intercostal space, respectively. The average distance from the edge of the CTV_RTOG_ and the edge of CTV_DBCG_ to the central points of the IM-SLNs was 4.10 mm (SD, 3.3 mm) and 1.60 mm (SD, 2.6 mm), respectively (*t* = 16.640, *P* = 0.000). The average distance from the edge of CTV_RTOG_ and the edge of CTV_DBCG_ to the lateral border IM-SLN was 6.40 mm (SD, 3.5 mm) and 3.34 mm (SD, 3.3 mm), respectively (*t* = 19.815, *P* = 0.000). Only 18.4% of IM-SLN central points were included in the CTV_RTOG_, and 60.3% of IM-SLN central points were included in the CTV_DBCG._ When covering 90 and 100% of the IM-SLN center points, the CTV_RTOG_ needs to expand 8 and 15 mm, respectively, and the CTV_DBCG_ needs to expand 5 and 13 mm, respectively.

**Conclusion:** Neither the RTOG nor DBCG consensus guideline about the delineation of IMN CTV was sufficient to cover 90% of IM-SLNs. For 90% coverage of IM-SLN central points, CTV_RTOG_ needed to be expanded by 8 mm, and CTV_DBCG_ needed to be expanded by 5 mm.

## Introduction

As the first-echelon nodal drainage site of breast cancer, IMN is one of the important metastatic ways of breast cancer (1). Due to the risk of cardiac (1, 2), prophylactic internal mammary node irradiation (IMNI) used to be controversial (3, 4). Recently, numerous randomized controlled trials have indicated that IMNI can improve local-control rate and overall survival (5–8). Prophylactic IMNI for high-risk breast cancer patients has drawn renewed attention.

The definition and delineation of optimal target volume are the key to radiotherapy. However, due to lack of sufficient data to determine what the exact area of the IMNs, contouring guidelines of IMNI are not identical (9–14). The existing guidelines of the IMNI were made based on the location of metastatic or recurrent IMNs, the position of internal mammary vessels, and surrounding anatomical structures. Actually, metastatic lymph nodes usually grow in an asymmetric and anisotropic manner, and the surrounding anatomical structure of the large metastatic lymph nodes may be changed. In contrast, with complete information on the anatomical and physiological structure, the IM-SLNs may be more suited to define the scope of prophylactic irradiation for IMNs. Even more important, the lymphoscintigraphy by SPECT/CT can provide functional and anatomical images in the same scanning session, and the anatomical position of IM-SLNs can be presented on the SPECT/CT combination images for every individual patient (15, 16).

This retrospective study explored the coverage rate of the consensus guidelines (RTOG and DBCG) by mapping the distribution of IM-SLNs presented on SPECT/CT images. The purpose was to explore the value of imaging IM-SLN on SPECT/CT to guide tailored CTV delineation of postoperative prophylactic IMNI.

## Materials and Methods

### Patients

A total of 709 breast cancer patients who underwent preoperative lymphoscintigraphy by SPECT/CT from April 2014 to April 2018 were enrolled, and 97 patients with 136 IM-SLNs were selected. The selected patients for this study should cover the following criteria, who should be first diagnosed breast cancer, whose IMNs on diagnosed MRI or CT images were negative, and whose positive IM-SLNs were detected on SPECT/CT images. The patients who accepted neoadjuvant treatment were excluded. The studies involving human participants were reviewed and approved by Shandong Tumor Hospital Ethics Committee. Due to the retrospective nature of the study, requirement for informed consent was waived.

### SPECT/CT Imaging

The SPECT/CT imaging used the 99mTc-labeled sulfur colloid as the tracer. Approximately 3–16 h before sentinel lymph node biopsy of the internal mammary chain (IM-SLNB), 18.5–37.0 MBq 99mTc-SC in volumes of 0.3–0.5 ml were injected into the mammary gland at 6 and 12 o'clock of the areola surrounding area.

**Figure 1:**
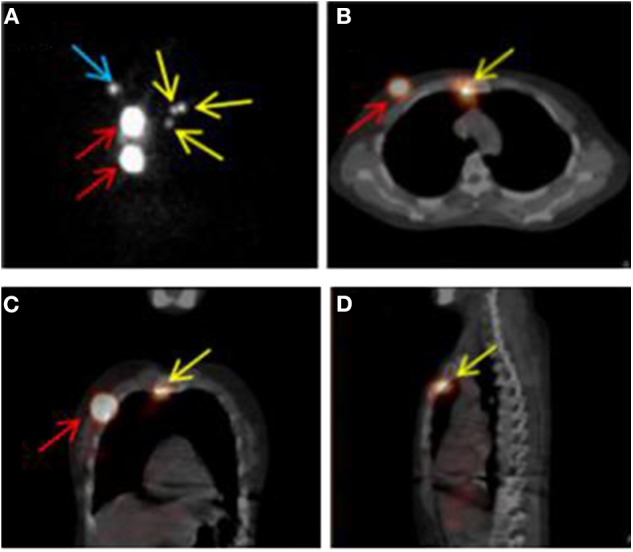
SPECT/CT combination imaging and positive sentinel lymph nodes. **(A)** SPECT image in anteroposterior position. **(B–D)** SPECT/CT fusion images. Red, yellow, and blue arrows indicate injection points, positive internal mammary sentinel lymph nodes, and axillary sentinel lymph node, respectively.

The SPECT/CT system (Philips Bright View XCT, Philips Healthcare, The Netherlands) includes a dual-head variable-angle gamma camera equipped with low-energy high-resolution collimators and a three-slice spiral CT scanner optimized for rapid rotation. Early planar scintigraphy was made at an average of 30 min after injection. Anterior and lateral position planar scintigraphy were taken with the patients in the supine position with both arms raised overhead. The planar scintigraphy was set as matrix, 256 × 256; zoom, 1; peak energy, 140 keV, and performed using steps of 6 degrees (10 s per view). SPECT/CT was made immediately after planar scintigraphy. CT scan was performed first, followed by SPECT imaging immediately. CT parameters were as follows: matrix, 512 × 512; thickness, 1 mm; peak energy, 120 keV; 20 mA; SPECT parameters were as follows: matrix, 64 × 64; peak energy, 140 keV; zoom 1.46 × (40.9) cm. Fused SPECT/CT was displayed using orthogonal multiplanar reconstruction and maximum intensity projection. Reconstructions were obtained in transversal, sagittal, and coronal planes. Except for the injection sites, the focal accumulations of radioactivity were identified as SLN (Figure 1).

### IM-SLN Delineation

A random patient who had undergone breast-conserving surgery was selected to be the simulated standardized patient. Her axial CT scan in the same posture as the SPECT/CT image was chosen as the representative CT image. The distributions of IM-SLNs in each intercostal space were recorded. The IM-SLNs presented on SPECT/CT images of the eligible patient were delineated, and the central point of the delineated target was marked as the central point of the IM-SLN. Using the deformation registration function of the MIM software (version 6.7.6), the SPECT/CT image of each patient was fused based on density with the representative CT image. Then, the center points of IM-SLNs were transferred from the SPECT/CT fusion images onto corresponding anatomical positions of the representative CT images. Previous studies have indicated that the average size of the IM-SLNs was 5 mm (17–19). All imaging IM-SLNs were plotted with a diameter of 5 mm, and named IM-SLN_5mm_.

### IMN CTV Delineation

**Figure 2:**
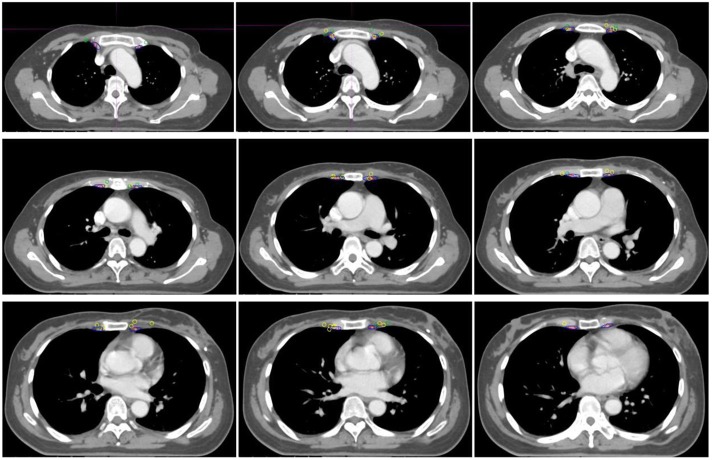
The position relationship between CTV_RTOG_, CTV_DBCG_, and IM-SLNs. Red, blue, dark green, and yellow circles indicate the CTV_RTOG_, CTV_DBCG_, solitary IMNs, and multiple IMNs, respectively.

According to the RTOG and DBCG guidelines, the CTVs were delineated on the representative CT scanning images of the standardized patient, and defined as CTV_RTOG_ and CTV_DBCG_, respectively (Figure 2). The distance from the border of CTV to central points of IM-SLN and the lateral edge of IM-SLN_5mmS_ were measured, respectively. Then, CTV_RTOG_ and CTV_DBCG_ were homogeneously enlarged 1 mm at a time until all the central points of IM-SLNs and the volumes of IM-SLN_5mm_s were included in the CTVs. After each extension of CTV, the number of the center points and IM-SLN_5mm_s in the CTV was recorded.

### Statistical Analysis

The distribution of IM-SLNs and the coverage performance were analyzed by descriptive statistics. The average distance from two CTVs to the central points of IM-SLNs and the border of IM-SLN_5mm_s was analyzed by paired *t*-tests. When *P* < 0.05, differences were considered significant. All analyses were performed using the SPSS statistical software, version 22.0.

## Results

### Patient Characteristics

Out of the total number of 709 enrolled patients, 587 patients had negative IM-SLN lymphoscintigraphy, 21 patients received neoadjuvant systemic treatment before surgery, and 4 patients had indistinct or incomplete clinical data. A total of 97 patients met the criteria and were included. The 97 recruited patients had 136 lymphoscintigraphy-positive IM-SLNs. On average, each patient had 1.4 IM-SLNs. Demographic and clinical–pathological characteristics of the 97 patients are shown in Table 1.

**Table 1:** Clinical characteristics of the 97 enrolled patients.

**Characteristic**	**Value**
Age (y)	
Median	46
Range	21–66
Primary tumor, *n* (%)	
Left	48 (49)
Right	49 (51)
Histopathologic type, *n* (%)	
IDC	73 (75.3)
ILC	1 (1.0)
DCIS	11 (11.3)
Mixed	10 (10.3)
Mucinous	2 (2.1)
Primary tumor location, *n* (%)	
Medial	28 (28.9)
Central	16 (16.5)
Lateral	53 (54.6)
T category, *n* (%)	
Tis	4 (4.1)
T1	45 (46.3)
T2	43 (44.3)
T3	3 (3.1)
T4	2 (2.1)
N category, *n* (%)	
N0	75 (77.3)
N1	19 (19.6)
N2	0 (0)
N3	3 (3.1)
N4	0 (0)
M category, *n* (%)	
M0	97 (100)
M1	0 (0)

### IM-SLN Mapping

**Figure 3:**
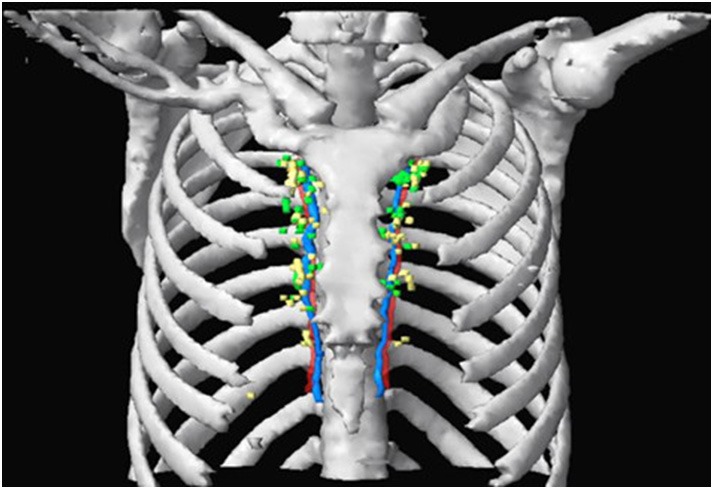
SPECT/CT fused with CT image showing IM-SLN distribution in different intercostal spaces. Green and yellow nodes indicate solitary IMNs and multiple IMNs, respectively. Red and blue indicate IM artery and IM vein.

Among 136 nodes, 65 were located on the left side of the sternum and 71 were located on the right. Overall, 40.4% of the IM-SLNs were located in the first intercostal space (*n* = 55), 30.2% in the second (*n* = 41), 24.3% in the third (*n* = 33), 4.4% in the fourth (*n* = 6), and 0.7% in the fifth (*n* = 1). The 136 IM-SLNs were mapped in Figure 3.

The average distance from the edge of the CTV_RTOG_ and the edge of CTV_DBCG_ to the central points of the IM-SLNs was 4.10 mm (SD, 3.3 mm) and 1.6 mm (SD, 2.6 mm), respectively (*t* = 16.640, *P* = 0.000). The average distance from the edge of CTV_RTOG_ and the edge of CTV_DBCG_ to the lateral border of IM-SLN was 6.4 mm (SD, 3.5 mm) and 3.34 mm (SD, 3.3 mm), respectively (*t* = 19.815, *P* = 0.000).

The coverage of center points of IM-SLNs and IM-SLN_5mm_s by different margin expansions of CTV_RTOG_ and CTV_DBCG_ is shown in Table 2. There were 25 (18.4%) center points and 7 (5.1%) IM-SLN_5mm_s located within CTV_RTOG_, while 82 (60.3%) center points and 38 (27.9%) IM-SLN_5mm_s were located within CTV_DBCG_. After the CTVs were homogeneously expanded by 1 mm each time, 70, 80, 90, and 100% of the center points were encompassed with a 5-, 6-, 8-, and 15-mm expansion of CTV_RTOG_, respectively. CTV_DBCG_ was expanded 2, 3, 5, and 13 mm to encompass 70, 80, 90, and 100% of the central points, respectively. To encompass 70, 80, 90, and 100% of the IM-SLN_5mm_s, CTV_RTOG_ should be expanded 8, 9, 11, and 17 mm, while CTV_DBCG_ should be expanded 5, 6, 7, and 16 mm, respectively.

**Table 2:** After different expansions of the CTV_RTOG_ and CTV_DBCG_, the coverage rate of the center point IM-SLNs and the whole volume IM-SLN.

**Expansion of the CTV** ** (mm)**	**CTV**_****RTOG****_		**CTV**_****DBCG****_	
	**Coverage of the Center point** ***n* (%)**	**Coverage of the IM-SLN** ***n* (%)**	**Coverage of the Center point** ***n* (%)**	**Coverage of the IM-SLN** ***n* (%)**
0	25 (18.4)	7 (5.1)	82 (60.3)	38 (27.9)
1	33 (24.3)	9 (6.6)	88 (64.7)	44 (32.4)
2	43 (31.6)	17 (12.5)	96 (70.6)	59 (43.4)
3	62 (45.6)	27 (19.9)	114 (83.8)	88 (64.7)
4	80 (58.8)	37 (27.2)	120 (88.2)	93 (68.4)
5	98 (72.1)	56 (41.2)	125 (91.9)	106 (77.9)
6	111 (81.6)	72 (52.9)	129 (94.9)	116 (85.3)
7	117 (86.0)	89 (65.4)	130 (95.6)	124 (91.2)
8	124 (91.2)	107 (78.8)	132 (97.1)	126 (92.6)
9	127 (93.4)	114 (83.8)	133 (97.8)	129 (94.9)
10	131 (96.3)	121 (89.0)	–	130 (95.6)
11	–	125 (91.9)	134 (98.5)	132 (97.1)
12	133 (97.8)	129 (94.9)	–	134 (98.5)
13	134 (98.5)	131 (96.3)	136 (100.0)	–
14	–	133 (97.8)	–	–
15	136 (100.0)	134 (98.5)	–	135 (99.3)
16	–	–	–	136 (100.0)
17	–	136 (100.0)	–	–

## Discussion

Most reported studies have reported the conformity between the distribution of metastatic or recurrence IMNs and the coverage of the IMNs by the CTV based on the consensus guidelines. As the first lymph node to receive drainage from the primary tumor, the position relationship between IM-SLNs and the IMN CTV has not been confirmed yet. IM-SLNB based on lymphoscintigraphy of SPECT/CT was a minimally invasive technique to evaluate the state of IM-SLN as well as to guide the individualized diagnosis and treatments for breast cancer (20–22). The radiotracer of lymphoscintigraphy can enter lymphatic capillaries via the gaps between cell junctions and the intercellular clefts formed by overlapping cells, followed by the lymphatic drainage, and finally gathered in the SLNs to form as a “hot spot” on SPECT image. The SPECT/CT was able to provide functional and anatomical information simultaneously, and locate IM-SLN more accurately (23). Based on these, in our current study, we try to find out the relationship between IM-SLN and consensus guidelines.

The basic principle to determine the target volume of irradiation for postoperative breast cancer patients is neither excessive nor inadequate. It is necessary for obtaining maximum tumor control while minimizing the toxicity of radiotherapy. However, the intercostal space delineation for IMNI CTV by different guidelines is significantly diverse. In the DBCG guideline, the caudal border of IMN CTV is the cranial edge of the fifth rib, while in the other guidelines, the limits of the caudal border is the cranial edge of the fourth rib (10–14). Except in a few cases, the first three intercostal spaces were always chosen to be the range of IMNI in clinical practice. The common location for the IM-SLN was the first three intercostal space. In addition, the recurrence and metastatic IM-SLN were also concentrated in the second and third intercostal space (24–26). In our study, 94.9% of IM-SLNs were located within the first three intercostal. The result was the same as the above studies. Therefore, combined with the intercostal distribution of IMN and IM-SLN, delineation of the first three intercostal spaces as the target of IM preventive radiotherapy is more reasonable.

A previous study has reported that both the lateral distance from the midsternum toward the same treatment side (range, 0.5–4 cm; median, 2.5 cm) and the lateral distance from the midline to the contralateral side (range, 0.75–1.6 cm; median, 1.5 cm) varied widely (27). Contouring guidelines (10–12, 14) that define the 5 mm to IM vessels as IMN CTV lateral border are all suggested, with the exception of the RTOG guidelines, which encompass only the IM artery and vein (13). In the study of Jethwa (28), the results showed that nodal metastases located medially and laterally from the IM vessels have a mean distance of 2.2 and 3.6 mm from the IM vessels, respectively. In our study, the mean distance between the edge of CTV_RTOG_ to the central points of IM-SLNs and the edges of IM-SLN_5mm_s was 4.10 and 6.40 mm, respectively. Compared with the CTV_RTOG_, the average distance between the central points of IM-SLNs and the edges of IM-SLN_5mm_s to the CTV_DBCG_ was smaller (1.60 mm, SD 2.6 mm, and 3.34 mm, SD 3.3 mm). Therefore, whether based on the literature reports or the results of this study, the lateral and medial boundary of CTV_RTOG_ and CTV_DBCG_ were significantly small. Besides, Borm et al. (29) found that in 55 positive IMNs presented on fluorine 18 fluorodeoxyglucose/computed tomography (18FDG-PET/CT), only two IMNs were completely within the described margins and a volume of 41.4 and 38.5% of the lymph nodes were included in the RTOG and ESTRO delineation, respectively. Our data showed that 18.4% of the central points and 5.1% of IM-SLN_5mm_s were located within the CTV_RTOG_, and 60.3% of the central points and 27.9% of IM-SLN_5mm_s were located within the CTV_DBCG_. On one hand, these results have once again shown that the IMN CTVs provided by the related guidelines were insufficient, and in order to cover the IMN more comprehensively, maybe the delineation for IMN CTV need to be optimized. On the other hand, it was indicated that maybe the current ranges of CTVs are unsuitable as the prophylactic irradiation region of IMNs; when making or updating the delineation for IMN CTV, the location of IM-SLN should be taken into more consideration.

With the exception of the RTOG guidelines, which encompass only the IM artery and vein (13), other guidelines suggested the IMN CTV include the IM vessels (IM vein and artery) with 5-mm margins (10–12). At present, the main methods for optimizing the target volume of RNI in various studies are based on the relationship between the metastatic or recurrent IMNs and the IM vessels. Davidson presented that a 5-mm medial and lateral margin expansion on the IMN CTV of the RTOG guidelines could include 93% of metastatic IMNs (30). In addition, according to the central points of IMNs, Jethwa et al. (28) considered that 90% of lymph nodes were encompassed with a 4-mm medial and lateral expansion on the RTOG IMN target volume. From the spatial distribution of IMN, only the lateral and medial extension of CTV may be insufficient. Our study used the IM-SLNs presented on SPECT/CT images and found that if only the central points of IM-SLNs were considered, the IMN CTV should be expanded by 8 and 5 mm based on the RTOG and DBCG guidelines, respectively. If the entire volume of IM-SLN (set as 5 mm in diameter) is considered, the CTV should be expanded by 11 and 7 mm from the CTV recommended by the RTOG and DBCG guidelines, respectively. The difference in results may indicate that the central points of metastatic lymph node may not necessarily represent the location of normal lymph nodes.

However, there are still some limitations in our study. Firstly, our respective study has a small sample size, and in a further study, more location information of IM-SLNs is needed to verify the results. Moreover, our study used the more professional image deformation registration technology instead of hand mapping, but the errors during transforming the location of IM-SLNs from the CT images of eligible patients to the standard patient were still inevitable.

In conclusion, neither the RTOG nor DBCG guidelines about IMN CTV delineation can provide a sufficient coverage of the IM-SLNs. Mapping the locations of IM-SLNs presented on the SPECT/CT could help to optimize the IMN CTV for breast cancer patients. In order to achieve a more comprehensive coverage of the IMN drainage for prophylactic irradiation, the location of IM-SLNs should be taken into account when evaluating and updating the above guidelines of delineation for IMN.

## Data Availability Statement

All datasets generated for this study are included in the article/supplementary material.

## Ethics Statement

The studies involving human participants were reviewed and approved by Shandong Tumor Hospital Ethics Committee. Due to the retrospective nature of the study, requirement for informed consent was waived.

## Author Contributions

XW contributed to the study design, the patient enrollment, the data statistics and analysis, and writing the manuscript. J-BL and WW participated in the study design and revising the content. Z-WH and MX contributed to reviewing the delineation. P-FQ, Y-JZ, F-XL, and J-ZW made important contributions in collecting the data. All authors read and approved the final manuscript.

### Conflict of Interest

The authors declare that the research was conducted in the absence of any commercial or financial relationships that could be construed as a potential conflict of interest.

## Abbreviations

IMN: internal mammary node
IMN: internal mammary node
IM-SLN: internal mammary sentinel lymph nodes
RTOG: Radiation Therapy Oncology Group
DBCG: Danish Breast Cancer Cooperative Group
IMNI: internal mammary node irradiation
IM-SLNB: sentinel lymph node biopsy of the internal mammary chain.

## References

[1] Hassiotou F Geddes D. Anatomy of the human mammary gland: current status of knowledge. Clin Anat. (2013) 26:29–48. 10.1002/ca.22165 22997014

[2] Cheng YJ Nie XY Ji CC Lin XX Liu LJ Chen XM. Long-term cardiovascular risk after radiotherapy in women with breast cancer. J Am Heart Assoc. (2017) 6:e005633. 10.1161/JAHA.117.005633 28529208 PMC5524103

[3] Choi J Kim YB Shin KH Ahn SJ Lee HS Park W. Radiation pneumonitis in association with internal mammary node irradiation in breast cancer patients: an ancillary result from the KROG 08-06 study. J Breast Cancer. (2016) 19:275–82. 10.4048/jbc.2016.19.3.275 27721877 PMC5053312

[4] Freedman GM Fowble BL Nicolaou N Sigurdson ER Torosian MH Boraas MC. Should internal mammary lymph nodes in breast cancer be a target for the radiation oncologist? Int J Radiat Oncol Boil Phys. (2000) 46:805–14. 10.1016/s0360-3016(99)00481-2 10705000

[5] Verma V Vicini F Tendulkar RD Khan AJ Wobb J Edwards-Bennett S. Role of internal mammary node radiation as a part of modern breast cancer radiation therapy: a systematic review. Int J Radiat Oncol Biol Phys. (2016) 95:617–31. 10.1016/j.ijrobp.2016.01.058 27131078

[6] Ragaz J Olivotto IA Spinelli JJ Phillips N Jackson SM Wilson KS. Locoregional radiation therapy in patients with high-risk breast cancer receiving adjuvant chemotherapy: 20-year results of the British Columbia randomized trial. J Natl Cancer Inst. (2005) 97:116–26. 10.1093/jnci/djh297 15657341

[7] Thorsen LB Offersen BV Danø H Berg M Jensen I Pedersen AN. DBCG-IMN: a population-based cohort study on the effect of internal mammary node irradiation in early node-positive breast cancer. J Clin Oncol. (2016) 34:314–20. 10.1200/JCO.2015.63.6456 26598752

[8] Whelan TJ Olivotto IA Parulekar WR Ackerman I Chua BH Nabid A Regional nodal irradiation in early-stage breast cancer. N Engl J Med. (2015) 373:307–16. 10.1056/NEJMc1510505 26200977 PMC4556358

[9] Stemmer SM Rizel S Hardan I Adamo A Neumann A Goffman J. The role of irradiation of the internal mammary lymph nodes in high-risk stage II to IIIA breast cancer patients after high-dose chemotherapy: a prospective sequential nonrandomized study. J Clin Oncol. (2003) 21:2713–8. 10.1200/JCO.2003.09.096 12860949

[10] Nielsen MH Berg M Pedersen AN Andersen K Glavicic V Jakobsen EH. Delineation of target volumes and organs at risk in adjuvant radiotherapy of early breast cancer: national guidelines and contouring atlas by the Danish Breast Cancer Cooperative Group. Acta Oncol. (2013) 52:703–10. 10.3109/0284186X.2013.765064 23421926

[11] Offersen BV Boersma LJ Kirkove C Hol S Aznar MC Biete Sola A. ESTRO consensus guideline on target volume delineation for elective radiation therapy of early stage breast cancer. Radiother Oncol. (2015) 114:3–10. 10.1016/j.radonc.2014.11.030 25630428

[12] Verhoeven K Weltens C Remouchamps V Mahjoubi K Veldeman L Lengele B. Vessel based delineation guidelines for the elective lymph node regions in breast cancer radiation therapy - PROCAB guidelines. Radiother Oncol. (2015) 114:11–6. 10.1016/j.radonc.2014.11.008 25466373

[13] Li XA Tai A Arthur DW Buchholz TA Macdonald S Marks LB. Variability of target and normal structure delineation for breast cancer radiotherapy: an RTOG Multi-Institutional and Multiobserver study. Int J Radiat Oncol Biol Phys. (2009) 73:944–51. 10.1016/j.ijrobp.2008.10.034 19215827 PMC2911777

[14] Dijkema IM Hofman P Raaijmakers CP Lagendijk JJ Battermann JJ Hillen B. Loco-regional conformal radiotherapy of the breast: delineation of the regional lymph node clinical target volumes in treatment position. Radiother Oncol. (2004) 71:287–95. 10.1016/j.radonc.2004.02.017 15172144

[15] Kraft O Havel M. Sentinel lymph nodes and planar scintigraphy and SPECT/CT in various types of tumours. Estimation of some factors influencing detection success. Nuclear Med Rev. (2013) 16:17–25. 10.1007/s12428-013-0013-0 23677759

[16] Vercellino L Ohnona J Groheux D Slama A Colletti PM Chondrogiannis S. Role of SPECT/CT in sentinel lymph node detection in patients with breast cancer. Clin Nucl Med. (2014) 39:431–6. 10.1111/lsq.12052 23877520

[17] Kinoshita T Odagiri K Andoh K Doiuchi T Sugimura K Shiotani S. Evaluation of small internal mammary lymph node metastases in breast cancer by MRI. Radiat Med. (1999) 17:189–93. 10440106

[18] Mack M Chetlen A Liao J. Incidental internal mammary lymph nodes visualized on screening breast MRI. AJR Am J Roentgenol. (2015) 205:209–14. 10.2214/AJR.14.13586 26102401

[19] Patel S Delikat A Liao J Chetlen AL. Pre- and post-magnetic resonance imaging features of suspicious internal mammary lymph nodes in breast cancer patients receiving neo-adjuvant therapy: are any imaging features predictive of malignancy? Breast J. (2018) 24:997–1000. 10.1111/tbj.13102 30066351

[20] Asadi M Krag D. Internal mammary sentinel lymph node biopsy in clinical practice. Int J Surg. (2016) 36(Pt A):332–4. 10.1016/j.ijsu.2016.11.034 27856352

[21] Giuliano AE Connolly JL Edge SB Mittendorf EA Rugo HS Solin LJ. Breast cancer-major changes in the american joint committee on cancer eighth edition cancer staging manual. CA Cancer J Clin. (2017) 67:290–303. 10.3322/caac.21393 28294295

[22] Bourre JC Payan R Collomb D Gallazzini-Crepin C Calizzano A Desruet MD. Can the sentinel lymph node technique affect decisions to offer internal mammary chain irradiation? Eur J Nucl Med Mol Imaging. (2009) 36:758–64. 10.1007/s00259-008-1034-4 19142635

[23] Borrelli P Donswijk ML Stokkel MP Teixeira SC van Tinteren H Rutgers EJ. Contribution of SPECT/CT for sentinel node localization in patients with ipsilateral breast cancer relapse. Eur J Nucl Med Mol Imaging. (2017) 44:630–7. 10.1007/s00259-016-3545-8 27787592 PMC5323474

[24] Qiu PF Cong BB Zhao RR Yang GR Liu YB Chen P. Internal mammary sentinel lymph node biopsy with modified injection technique: high visualization rate and accurate staging. Medicine. (2015) 94:e1790. 10.1097/MD.0000000000001790 26469922 PMC4616776

[25] Leidenius MH Krogerus LA Toivonen TS Leppänen EA von Smitten KA. The clinical value of parasternal sentinel node biopsy in breast cancer. Ann Surg Oncol. (2006) 13:321–6. 10.1245/ASO.2006.02.022 16485152

[26] Bi Z Chen P Liu J Liu Y Qiu P Yang Q. Internal mammary sentinel lymph node biopsy after neoadjuvant chemotherapy in breast cancer. J Breast Cancer. (2018) 21:442–6. 10.4048/jbc.2018.21.e49 30607166 PMC6310727

[27] Lotayef MM Barsoum MS Zaki OE Nasr MA Abdel Aziz RA Koteb M. Planning of the internal mammary field based on lymphoscintigraphy localization before postoperative radiotherapy of breast cancer. J Egypt Natl Canc Inst. (2005) 17:203–10. 16799658

[28] Jethwa KR Kahila MM Hunt KN Brown LC Corbin KS Park SS. Delineation of internal mammary nodal target volumes in breast cancer radiation therapy. Int J Radiat Oncol Biol Phys. (2017) 97:762–9. 10.1016/j.ijrobp.2016.11.037 28244412

[29] Borm KJ Voppichler J Düsberg M Oechsner M Vag T Weber W. FDG/PET-CT-based lymph node atlas in breast cancer patients. Int J Radiat Oncol Biol Phys. (2019) 103:574–82. 10.1016/j.ijrobp.2018.07.2025 30118822

[30] Davidson T Ben-David M Galper S Haskin T Howes M Scaife R. Use of 18F-FDG PET-CT imaging to determine internal mammary lymph node location for radiation therapy treatment planning in breast cancer patients. Pract Radiat Oncol. (2017) 7:373–81. 10.1016/j.prro.2016.11.001 28989000

